# Design and Simulation Study of a CNT-Based Multisource Cubical CT System for Dynamic Objects

**DOI:** 10.1155/2018/6985698

**Published:** 2018-08-30

**Authors:** Changcheng Gong, Li Zeng, Chengxiang Wang, Lei Ran

**Affiliations:** ^1^Key Laboratory of Optoelectronic Technology and Systems of the Ministry of Education of China, Chongqing University, Chongqing 400044, China; ^2^Engineering Research Centre of Industrial Computed Tomography Nondestructive Testing of the Ministry of Education of China, Chongqing University, Chongqing 400044, China; ^3^College of Mathematics and Statistics, Chongqing University, Chongqing 401331, China; ^4^College of Mathematical Sciences, University of Electronic Science and Technology of China, Chengdu 611731, China; ^5^College of Mechanical Engineering, Chongqing University, Chongqing 400030, China

## Abstract

The purpose of this paper is to design and simulate a new computed tomography (CT) system with a high temporal resolution for dynamic objects. We propose a multisource cubical CT (MCCT) system with X-ray tubes and detectors installed on a cube. Carbon nanotube- (CNT-) based X-ray focal spots are distributed on the twelve edges of the cube. The distribution of X-ray focal spots and detectors completely avoids mechanical movements to scan an object under inspection. CNTs are excellent electron field emitters because the use of a “cold” cathode makes it possible to fabricate a cathode with multiple electron emission points, and the CNT-based X-ray focal spots possess little response time and programmable emission. The proposed rotation-free MCCT system can acquire a high scanning speed when using a high frame rate detector. A three-dimensional (3D) reconstruction algorithm with tensor framelet-based L0-norm (TF-L0) minimization is developed for the simulation study of the MCCT. Simulation experiment results demonstrate the feasibility of the MCCT system.

## 1. Introduction

Computed tomography (CT) is an important nondestructive testing tool which has been widely applied to medicine, industry, biology, and so on [1–5]. In the field of medicine and biology, dynamic objects such as small animals and human hearts are the subjects most often investigated. It is known that cardiovascular disease is a serious menace to human health. On this account, small-animal imaging plays an important role in providing a basic understanding of the mechanism of the disease, drug discovery, clinical assessment, and so on [1]. However, physiological motions of small animals such as a mouse are much faster than those of humans, and motion-induced artifacts inevitably degrades the CT images [6]. CT and other imaging modalities have revolutionized clinical diagnosis. However, dynamic CT is practically difficult because of the requirements for high spatial resolution and temporal resolution. The challenge in scanning a dynamic object is the lack of real-time detection technology to monitor the rapid changes and alleviate the motion-induced artifacts.

To abate the motion-induced artifacts, a natural idea is to accelerate the scanning process. Since CT was introduced to clinical medicine, some researchers focused on increasing the temporal resolution using traditional CT systems. Previous studies suggested that the temporal resolution should not exceed 50 msec for cardiac CT [7]. For a single-source CT system, the source and the detector installed on a gantry simultaneously rotate around the object under inspection to acquire complete projections. The limitation in rotation speed makes it difficult to further increase the temporal resolution, though slip-ring technology greatly reduces the scanning time dominated by interscan delays [8].

Multisource CT can be used to improve the temporal resolution. In 1979, the first multiple-source CT system for dynamic objects was designed at the Mayo Clinic [9], consisting of a large number of X-ray sources and fluorescent screens. This idea has a profound impact on CT. Liu et al. [10] proposed a three-source and five-source micro-CT system and applied it for a half-scan Feldkamp-type reconstruction, which can deliver a 2.33 and 3.18 speedup factor, respectively. Kachelrieß et al. [11] suggested a spiral CT with *G* X-ray sources and detectors for cardiac imaging. An approximate cone-beam algorithm was used for CT reconstruction. The temporal resolution is roughly a factor of *G* better than that with a single-source CT scanner. In 2005, Siemens designed a dual-source CT system which produces high temporal resolution images without the loss of spatial resolution and halves the scanning time [12, 13]. Liu et al. [14] proposed a multisource X-ray interior imaging system to achieve an ultrafast scan for small animal imaging. This system indicated that a 50 msec scan speed is possible without gating at low dose. Wu et al. [15] proposed a swinging multisource CT system for aperiodic dynamic imaging in industrial applications.

All the multisource CT systems cited above employed a conventional hot-cathode X-ray tube which emits electrons when the filament is heated to over 1000 degrees Celsius. There are some limitations for the conventional X-ray tube with a “hot” filament. First, high working temperature and power consumption leads to the fact that the conventional X-ray source only contains one hot cathode. This design makes the projection data to be acquired by the rotation of either the X-ray tube/detector pair or the specimen under inspection. Second, thermionic emission has a time delay which is not conducive to the improvement of the temporal resolution. These features make it difficult to acquire a high temporal resolution using conventional X-ray sources. Compared to the X-ray tube with a hot-cathode, a carbon nanotube- (CNT-) based X-ray tube has some advantages such as a compact structure, high temporal resolution, and programmable emission. In recent years, the CNT-based X-ray source has been greatly developed [16–18]. The voltage instead of resistive heating is used to control the tube current [18]. Due to the low power consumption and low working temperature of the carbon nanotube field emission cathode [17], multiple electron emission points can be integrated into one cathode, which leads to multiple X-ray focal spots. Then, miniature X-ray sources become possible [18, 19]. Some CT systems have been proposed to investigate possible applications of the CNT-based X-ray source. Hadsell et al. [20] designed a compact microbeam radiation therapy system using a CNT-based X-ray source. A long focal line was used to dissipate heat on a stationary anode, then a high-flux X-ray can be acquired from the focal line. Gonzales et al. [21, 22] proposed a rectangular CT scan setup focusing on security applications, which is a stationary CT system based on CNT X-ray tubes. This CT system uses linear CNT X-ray tubes to construct a stationary CT setup and the CNT-based X-ray source contains a dense array of independently controlled electron emission points. Zhao et al. [23] proposed a helical interlaced-source-detector-array CT, which can provide ultrahigh temporal resolution without any mechanical motions. Approximately complete projections were acquired through interpolations. However, complete projection data usually corresponds to high radiation dose. Cao et al. [24] proposed a stationary-source rotating-detector CT architecture to increase the temporal resolution and reduce the radiation dose. However, the rotating detector may reduce the temporal resolution.

**Figure 1:**
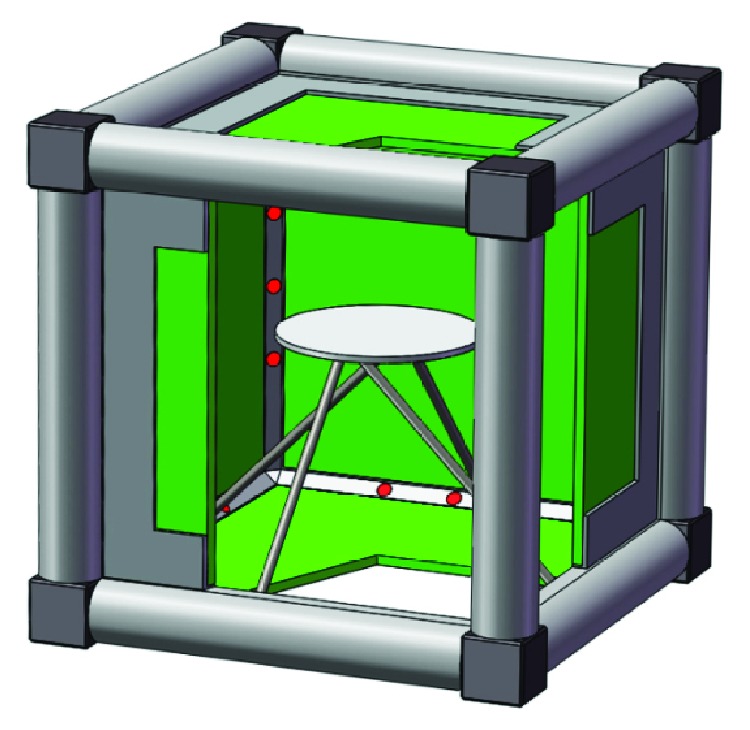
Illustration of a MCCT configuration. The illustration is cut open for display. The green components represent the detectors, the red points represent the focal spots in the CNT-based X-ray tube, and the white component is the objective table.

Motivated by the aforementioned facts, we propose a multisource cubical CT (MCCT) system with multiple CNT-based X-ray sources (Figure 1) to improve the temporal resolution for dynamic objects. The MCCT is an initial proof-of-concept design which could help us to understand some difficulties of CT system design. The MCCT also has some differences compared to other multisource CT systems.

The first difference in the MCCT is that the sources and detectors are fixed in the “stationary” MCCT. 12 X-ray sources and 6 flat-panel detectors constitute the cube shape with the merits of a compact structure and portability. The rotation of the X-ray source is replaced by instantly switching on and off different focal spots. No rotation mechanism/slip rings are required. The gantry is only used to support the cubical configuration. Taken together, the rotation-free MCCT avoids the artifacts caused by mechanical movements. In the traditional CT system, the rotation of the detector is commonly synchronized with the X-ray source to collect X-ray photons. In the MCCT, the “closed” structure enables the detectors to collect X-ray photons from all directions. Furthermore, the MCCT system is designed for small animals or other potential dynamic objects, so the tested objectives are relatively fixed; the system parameters of the MCCT could be optimized to favour high temporal resolution and high image quality.

The second difference in the MCCT is that 12 X-ray sources are installed on the edges of the “cubical” CT system, which can integrate multiple electron emission points without restricting the imaging region that happens in other multisource CT systems [10, 14, 15, 24]. For example, in the multisource X-ray interior imaging system [14], there are *K* (odd number) X-ray tube/detector pairs mounted on two coaxial circular trajectories in a plane. The detector may block some X-rays from the X-ray source on the opposite side. An odd number *K* is used to avoid the congestion of the CT equipment, which will reduce the field of view (FOV). A balance between the temporal resolution and the size of the FOV must be considered carefully.

The third difference in the MCCT is that the CNT-based X-ray source needs very little time to respond and has electronic programmability, fast switching, miniature size, and so on [25], which enables us to equate approximately the scanning time with exposure time. Thus, the temporal resolution can be enormously improved. We can also improve the temporal resolution of a CT system using electron beam computed tomography (EBCT) whose operation mode is similar to the CNT-based CT system [26]. However, the dimension of the X-ray source in EBCT is too large to integrate and the viewing angle range is limited by the difficulty of operating a high-energy electron beam.

 The fourth difference in the MCCT is that complete projection data can be acquired if the electron emission points are densely distributed in the X-ray source. However, acquiring complete projections may produce more radiation doses and lower the temporal resolution. In practice, each focal spot occupies a certain space and some space exits between two adjacent focal spots. Thus, “sparse” sampling may be a good choice for the MCCT, which can save the scanning time and lower the potential radiation dose. The so-called sparse sampling is performed on the trajectory formed by the twelve edges of the cube instead of the circular or spiral trajectory. The possible drawback is that the special sampling may lead to some artifacts looking like limited-angle artifacts when the positions of the electron emission points are not spread evenly, and reasonable prior knowledge must be integrated into the reconstruction model to constrain the solution.

Some iterative methods [27–34] have been proposed to deal with the CT reconstruction with incomplete projection data. For instance, total variation (TV) minimization can be used to reduce streak artifact and noise. A wavelet tight frame can provide a sparse representation with redundant information. In particular, tensor framelet (TF) is superior to the wavelet tight frame for high-dimensional image reconstruction in the sense of memory requirement and computational cost [35]. For 3D image reconstruction, tensor framelet is our preference. To enhance the sparsity of the reconstructed image, L0-norm minimization is used in our reconstruction task.

The structure of this article is as follows: in Section 2, we describe the geometric structure of the CT setup and the image reconstruction method TF-L0 is introduced in detail. Then, the simulation results are reported in Section 3. Finally, we discuss relevant issues and conclude this work in Section 4.

## 2. Materials and Methods

**Figure 2:**
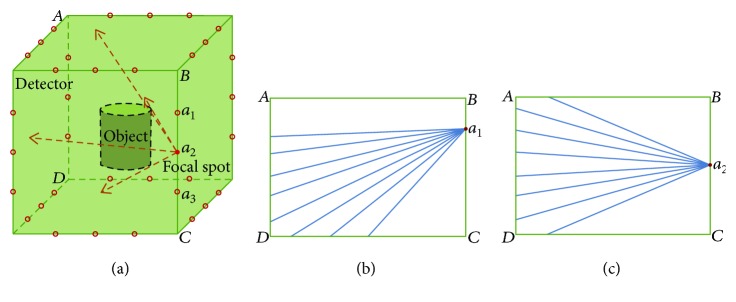
(a) Diagrammatic sketch of the MCCT with multiple X-ray focal spots. (b, c) The shapes of the collimated X-ray cone beam emitted from some different focal spots are different.

Geometry of the designed multisource cubical CT (MCCT) is shown in Figure 2. The MCCT is mounted with 12 X-ray sources and 6 flat-panel detectors. Each X-ray tube occupies one edge of the cube; 6 detectors constitute the 6 faces of the cube. The focal spots on one edge are sealed in a vacuum chamber with *n* X-ray exit windows. A natural concern is that the X-ray tube may create large gaps between two detectors, since the vacuum chamber is large. Then, these gaps become a major problem in terms of data completeness and CT reconstruction. As we know, each focal spot corresponds to an X-ray exit window. Thus, the main part of the designed X-ray tube gives way to these X-ray exit windows to minimize the data loss (Figure 1). Specifically, the main part of the X-ray tube is designed at the outboard of the MCCT system so that the X-ray exit windows are just located at the junction between two detectors. As a result, the missing data on the virtual detector are not as much as intuitively seen.

The objective table is supported in the central area of the CT. The material of the object table is strong, and the attenuation coefficient is very small. When the system is actually set up, one detector is designed to be attachable to the rest modules of the MCCT; other modules are mounted on an optical bench. The object can be loaded into the cubic scanner before the detachable detector is attached to the MCCT. If the object is a live small animal, this small animal would first be anesthetized by a professional experimenter. Then, the anaesthetized animal would be placed in a flat-bottomed box with its limbs fixed on the box. Then, the box is placed on the object table. Finally, the detachable detector would be attached to the MCCT. During the rapid CT scan, we can use an infrared pinhole camera to monitor the anaesthetized animal. The space for the camera is designed to be located near some X-ray exit window so the camera will not introduce additional projection data loss. Nevertheless, data loss is inevitable. We will simulate these data losses caused by the X-ray exit windows in Section 3.1.

As shown in Figure 2, the dots represent the X-ray focal spots. There are *n* focal spots linearly placed on each edge providing a total number of 12 *n* focal spots. For convenience, we assume that *n* focal spots are symmetrically distributed on each X-ray tube and we evenly spread the positions of the focal spots to increase the cover area. As shown in Figure 2(a), the focal spot indicated by the red dot *a*_2_ emits the collimated X-ray cone beam. The X-ray photons will be collected by four opposing detectors. In this work, projections at sparse views are collected to reduce the scanning time; thus, only a small number of the focal spots are needed.

**Figure 3:**
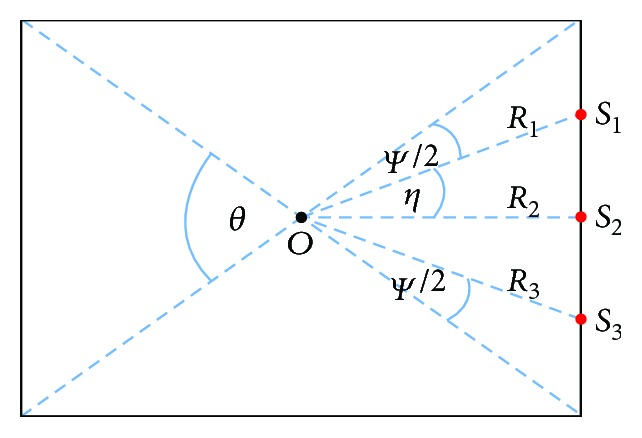
Graphical representation of the parameters for the MCCT.

As shown in Figure 3, the angle between two adjacent projection views is equivalent and assumed to be *η*. Denote $\theta =2\arctan\left(\sqrt{2}/2\right)\left(\approx 7\pi /18\right)$ the angle range an edge covers. Let *ψ* represent the angle range which is not covered by focal spots. For simplicity, we assume that *ψ* = *θ* − *π*/3. Then, the angle *η* can be determined by *γ* and the number of focal spots *n* : *η* = (*θ* − *ψ*)/(*n* − 1) = *π*/3(*n* − 1). For the special case of *n* = 3, *a*_1_, *a*_2_, and  *a*_3_ represent three focal spots. The distance from the current focal spot to the centre of the cube is *l*_1_, *l*_2_, and  *l*_3_, respectively, which can be calculated by simple geometric relations. The mathematical expression for each focal spot can be expressed using the aforementioned information. For instance, the respective coordinates of focal spots *a*_1_, *a*_2_, and  *a*_3_ are

(1) $$\begin{array}{c} c_{1}=\left(\frac{L}{2},\frac{L}{2},l_{2}\cdot \tan\left(\eta \right)\right),\\ c_{2}=\left(\frac{L}{2},\frac{L}{2},0\right),\\ c_{3}=\left(\frac{L}{2},\frac{L}{2},-l_{2}\cdot \tan\left(\eta \right)\right). \end{array}$$

We consider an object represented by a function *f*(*c*) with the coordinate *c* = (*x*, *y*, *z*) ∈ *R*^3^. Then, the projection can be calculated as follows:

(2) $$\begin{array}{c} p\left(c,d\right)=\int_{0}^{L\left(u,v\right)}f\left(c+\zeta d\right)d\zeta , \end{array}$$

where **d** = (*d*_1_, *d*_2_, *d*_3_) is the X-ray direction vector, (*u*, *v*) is the coordinate of the detector unit, *L*(*u*, *v*) is the length of the X-ray line, and *ζ* denotes the integration variable. CT reconstruction is to recover the distribution of the attenuation coefficients *f*(*c*) from the projections *p*(*c*, **d**). One CT scan for a dynamic object can be completed quickly, and the CT image at this moment is reconstructed using currently available projections.

**Figure 4:**
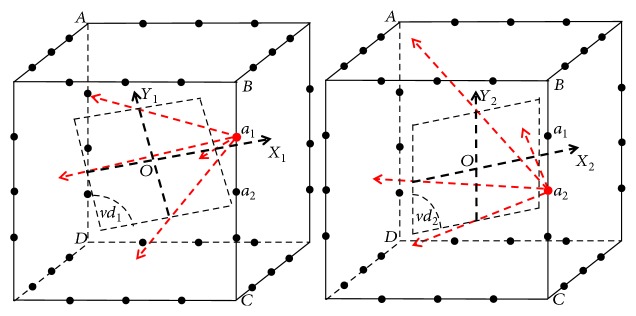
Two typical virtual detectors (*vd*) and diagrams of two imaging geometries for different focal spots.

We have to point out that the shape of the X-ray cone beam may be different for different views due to the closure of the MCCT and the linear distribution of the focal spots on the special trajectory (Figure 2). In practical applications, the measured projection data should be rebinned for CT reconstruction. In the following simulation experiments, the projections in the virtual detector (*vd*) are used for CT reconstruction. For each focal spot, there is an opposing virtual detector as shown in Figure 4. Two orthogonal directions in the virtual detector and the direction of the focal spot can constitute a Cartesian coordinate system. For example in Figure 4, *O* − *X*_1_*Y*_1_*a*_1_ and *O* − *X*_2_*Y*_2_*a*_2_ are two different Cartesian coordinate systems for focal spots *a*_1_ and *a*_2_. We can see that the distance between *a*_1_ and *O* is not equal to the distance between *a*_2_ and *O*. The position of virtual detector *vd*_1_ is different from the position of virtual detector *vd*_2_. Besides, the object *f* has different vector forms in *O* − *X*_1_*Y*_1_*a*_1_ and *O* − *X*_2_*Y*_2_*a*_2_. In the simulation experiments for the MCCT, the distance from the focal spot to the virtual detector and the position of the virtual detector will lead to different imaging geometries. For example in *O* − *X*_1_*Y*_1_*a*_1_, the projection can be calculated based on the geometry determined by the position of the focal spot *a*_1_ and the virtual detector *vd*_1_. Specifically, there are three steps to calculate the projection:

- Determine the distance from the focal spot *a*_1_ to the virtual detector *vd*_1_.
- Acquire the vector form of the object in the Cartesian coordinate system*O* − *X*_1_*Y*_1_*a*_1_.
- Calculate the projection by a line integral similar to (2).

**Figure 5:**
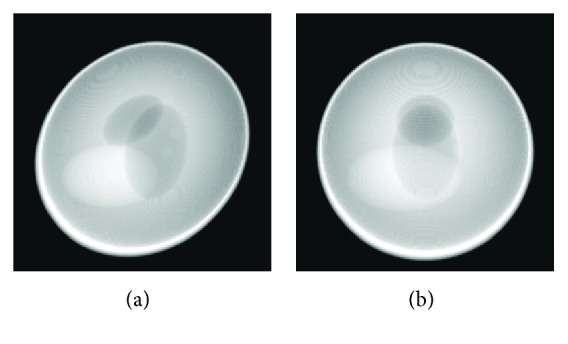
Two typical projection images for different focal spot.

The projection operators are different for different focal spots. Two typical projection images of the 3D Shepp-Logan phantom are shown in Figure 5. The simulated projections at other focal spots can be calculated in a similar way.

The CNT-based X-ray source consists of a field emission cathode with multiple electron emission points, a gate electrode, a focusing unit, and an anode packaged inside a vacuum chamber. During a CT scan, the field emission cathode emits electrons under the effect of the gate voltage. Then, the electrons are focused on the specific position on the anode by the focusing unit, which leads to multiple focal spots on the anode. The switch of the electron emission can be controlled by adjusting the gate voltage. Instantly switching on and off different focal spots is used to replace the rotation of the X-ray source/detector pair. Then, the X-ray cone beams are collimated to the central region of the MCCT. The energies of X-rays attenuated by the dynamic object are also recorded by the detectors.

Because the CNT-based X-ray source has electronic programmability and little response time, the exposure time is approximately equal to the scanning time for each projection view. The total scanning time *t*_total_ of the MCCT can be determined by the number of the projection views, the exposure time *t*_*e*_, and the detector acquisition time *t*_*a*_. The detectors are commonly synchronized with the X-ray sources to collect X-ray photons. Thus, the exposure time *t*_*e*_ is approximately equal to the acquisition time *t*_*a*_. With appropriate photon flux, exposure time can be reduced without degrading the image quality. Therefore, the proposed MCCT has the potential to improve the temporal resolution.

### 2.1. Discretized Representation of a CT Imaging System

CT imaging model can be approximated by a linear system:

(3) $$\begin{array}{c} g=Af. \end{array}$$

In practice, the acquired projections are discrete. Thus, **g** denotes the projection data vector. For 3D CT imaging, the object function (X-ray attenuation coefficient) *f* is the vectorized form of the 3D volume and *A* is the projection operator. The image reconstruction problem is to find *f* from the projection data **g** by solving the linear system equation.

The proposed MCCT has a special cube shape, and the trajectory of the X-ray focal spots is on the edges of the cube. This new trajectory is different from a conventional circular or spiral trajectory. Besides, only a small number of focal spots can be integrated on one edge because of the limited space within each X-ray tube. Thus, “sparse” sampling on the new trajectory can be used to acquire projections. This sampling is approximately sparse in 3D space formed by the cube. Thus, a traditional analytic reconstruction algorithm is not suitable for this CT reconstruction. Due to these differences, it is necessary to research the iterative reconstruction algorithm.

### 2.2. TV-Based Reconstruction Algorithm

Usually, an underdetermined problem appears when only projection data at sparse views are available. Thus, prior knowledge has to be used to regularize the solution *f*. The major idea of total variation (TV) minimization is that the signal can be reconstructed from few samplings if the signal is sparse in the gradient domain. TV of an image is defined as [27]

(4) $$\begin{array}{c} TV\left(f\right)=\underset{i,j,k}{\sum }\sqrt{{\left(f_{i,j,k}-f_{i-1,j,k}\right)}^{2}+{\left(f_{i,j,k}-f_{i,j-1,k}\right)}^{2}+{\left(f_{i,j,k}-f_{i,j,k-1}\right)}^{2}}. \end{array}$$

Then, the TV-based algorithm is utilized to solve the following optimization problem:

(5) $$\begin{array}{c} \min\;TV\left(f\right)\\ \text{Subject}\;\;\text{to}\;Af=g,\;f\geq 0. \end{array}$$

The solution of (5) can be acquired by an alternative minimization method consisting of two phases: projection on convex set step and gradient descent step.

### 2.3. L0-Norm Minimization-Based Reconstruction Algorithm

For 2D imaging, the memory cost is acceptable for multilevel wavelet tight frame transform. However, the memory cost is enormous for high-dimensional large-scale image reconstruction. Tensor framelet (TF) is better than the wavelet framelet in the demand on the memory and computational cost [35]. The TF transform denoted by *W* with its adjoint *W*^*T*^ has the feature *W*^*T*^(*Wf*) = *f*. In this work, we use the piecewise-linear framelet basis. The 1D piecewise-linear framelet basis functions are approximated by three refinement masks:

(6) $$\begin{array}{c} h_{0}=\frac{1}{4}\left[\begin{array}{ccc} 1 & 2 & 1 \end{array}\right],\\ h_{1}=\frac{\sqrt{2}}{4}\left[\begin{array}{ccc} 1 & 0 & -1 \end{array}\right],\\ h_{2}=\frac{1}{4}\left[\begin{array}{ccc} 1 & 2 & -1 \end{array}\right]. \end{array}$$

For a given image, convoluting with different refinement masks generates different frequency components. In [36], the L0-norm-based image reconstruction algorithm is proposed for 2D CT reconstruction. L0 norm could better characterize the sparsity and protect image edges. We generalize this algorithm to 3D CT reconstruction for the proposed MCCT. Then, 3D piecewise-linear framelet basis functions used in this work can be constructed by the tensor product of the 1D basis functions. The reconstruction model can be formulated as follows:

(7) $$\begin{array}{c} \underset{f\geq 0}{\arg\min}\left\{\frac{1}{2}{\left\|Af-g\right\|}_{2}^{2}+\lambda {\left\|Wf\right\|}_{0}+\frac{\gamma }{2}{\left\|f\right\|}_{2}^{2}\right\}, \end{array}$$

where *λ* and *γ* are scalar parameters, and ‖·‖_0_ denotes the L0 quasinorm. The first term is the data fidelity term to enhance data consistency. The second term makes the reconstructed image sparse. The term *γ*/2‖*f*‖_2_^2^ intends to reduce the energy of the reconstructed image and makes the reconstructed image smoother. Then, an auxiliary variable *α* is introduced with *α* = *Wf*, which is converted into the following problem by penalizing the equality constraint:

(8) $$\begin{array}{c} \underset{f\geq 0,\alpha }{\arg\min}\left\{\frac{1}{2}{\left\|Af-g\right\|}_{2}^{2}+\lambda {\left\|\alpha \right\|}_{0}+\frac{\tau }{2}{\left\|Wf-\alpha +\nu \right\|}_{2}^{2}+\frac{\gamma }{2}{\left\|f\right\|}_{2}^{2}\right\}, \end{array}$$

where *ν* is used for error feedback. The splitting technique is used to deal with (8) and the iterations are as follows:

(9) $$\begin{array}{c} f^{\left(t+1\right)}=\underset{f\geq 0}{\arg\min}\left\{\frac{1}{2}{\left\|Af-g\right\|}_{2}^{2}+\frac{\tau }{2}{\left\|Wf-\alpha +\nu \right\|}_{2}^{2}+\frac{\gamma }{2}{\left\|f\right\|}_{2}^{2}\right\},\\ \alpha^{\left(t+1\right)}=\underset{\alpha }{\arg\min}\left\{\lambda {\left\|\alpha \right\|}_{0}+\frac{\tau }{2}{\left\|Wf-\alpha +\nu \right\|}_{2}^{2}\right\},\\ \nu^{\left(t+1\right)}=\nu^{\left(t\right)}-\left(\alpha^{\left(t+1\right)}-{Wf}^{\left(t+1\right)}\right). \end{array}$$

Due to the huge memory cost of the system matrix **A**, the proximal point technique can be used to solve the problem efficiently [37]. Then, the data fidelity term can be linearized at a current point *f*^(*t*)^:

(10) $$\begin{array}{c} \frac{1}{2}{\left\|Af-g\right\|}_{2}^{2}=\frac{1}{2}{\left\|{Af}^{\left(t\right)}-g\right\|}_{2}^{2}+\langle f-f^{\left(t\right)},A^{T}\left(Af-g\right)\rangle +\frac{\beta }{2}{\left\|f-f^{\left(t\right)}\right\|}_{2}^{2}, \end{array}$$

where *β* is a parameter. Substitute (10) to the first step in (9) and then reformulate it in terms of square, we have the following:

(11) $$\begin{array}{c} \underset{f\geq 0}{\arg\min}\left\{\frac{1}{2}{\left\|f-\left(f^{\left(t\right)}-\frac{1}{\beta }A^{T}\left({Af}^{\left(t\right)}-g\right)\right)\right\|}_{2}^{2}+\frac{\tau /\beta }{2}{\left\|Wf-\alpha +\nu \right\|}_{2}^{2}+\frac{\gamma /\beta }{2}{\left\|f\right\|}_{2}^{2}\right\}. \end{array}$$

Approximately, a proximal point *f*^(*t* − *temp*)^ is found by an iteration method similar with the simultaneous algebraic reconstruction technique (SART). For simplicity, we also call this method SART. Therefore, (11) can be rewritten as:

(12) $$\begin{array}{c} \underset{f\geq 0}{\arg\min}\left\{\frac{1}{2}{\left\|f-f^{\left(t-temp\right)}\right\|}_{2}^{2}+\frac{\tau /\beta }{2}{\left\|Wf-\alpha +\nu \right\|}_{2}^{2}+\frac{\gamma /\beta }{2}{\left\|f\right\|}_{2}^{2}\right\}. \end{array}$$

Based on the above derivation, the iterative reconstruction algorithm can be described as follows:

(a) Initialization: *ε*_1_, *ω* > 0, *τ* > 0, *γ* = 1, *f*^(1)^ = 0, *α*^(1)^ = *ν*^(1)^ = *Wf*^(1)^, *ε* = 1, and  *t* = 1
(b) SART for a proximal point: *f*_*n*_^(*t* − *temp*)^ = *f*_*n*_^(*t*)^ + 1/*β ***A**^*T*^(**g** − *Af*_*n*_^(*t*)^),  *t* = 1, 2,…, *N*_*ite*_
(c) Minimization of (12): *f*^(*t* + 1)^ = (*f*^(*t* − *temp*)^ + (*τ*/*β*) · *W*^*T*^(*α*^(*t*)^ − *ν*^(*t*)^))/(1 + *τ*/*β* + *γ*/*β*)
(d) Positivity constrain: *f*_*n*_^(*t* + 1)^ = max(*f*_*n*_^(*t* + 1)^, 0)
(e) Iterative hard threshold (IHT) step: $\lambda >0,\;\;\tilde{\lambda }=\sqrt{2\lambda /\tau },\;\;\alpha^{\left(t+1\right)}=H_{\tilde{\lambda }}\left({Wf}^{\left(t+1\right)}+\nu^{\left(t\right)}\right)$; where
  (13) $$\begin{array}{c} H_{\tilde{\lambda }}\left(x\right)=\left\{\begin{array}{cc} 0 & \left|x\right|<\tilde{\lambda }\\ \left\{0,x\right\} & \left|x\right|=\tilde{\lambda }\\ x & \left|x\right|>\tilde{\lambda } \end{array}\right. \end{array}$$
(f) Update the Lagrangian multiplier and initialize the next loop: *ν*^(*t* + 1)^ = *ν*^(*t*)^ − (*α*^(*t* + 1)^ − *Wf*^(*t* + 1)^); *ε* = ‖*f*^(*t* + 1)^ − *f*^(*t*)^‖_2_/‖*f*^(*t* + 1)^‖_2_; and *γ*^(*t* + 1)^ = 0.9 × *γ*^(*t*)^

Increase *t* and go to step (b). The total number of iterations is *N*_*ite*_. The iteration is stopped when *ε* is smaller than the threshold value *ε*_1_ or *t* > *N*_*ite*_. We call this iteration algorithm TF-L0 for convenience.

Compared to the original algorithm in [36], there are some differences in the presented TF-L0. First, the TF-L0 is designed for 3D imaging on a special trajectory determined by the twelve edges of the cubical CT instead of 2D imaging on a circular trajectory. Second, the tensor frame transform is utilized in this work to save memory and computational cost, while wavelet tight frame transform was previously used as the sparse transform. Third, in this work, step (c) is solved analytically because of the differentiability of the cost function and the property *W*^*T*^(*Wf*) = *f*, while an iterative method algorithm was used to deal with step (c) iteratively in the original algorithm.

## 3. Results and Discussion

The results of the simulation experiments are presented as a proof of concept for the MCCT. The following iterative reconstruction methods are performed: SART, TV, and TF-L0. To quantify the reconstruction results, root mean square error (RMSE) and peak signal-to-noise ratio (PSNR) are calculated as follows:

(14) $$\begin{array}{c} RMSE=\sqrt{\frac{1}{N}\overset{N}{\underset{p=1}{\sum }}{\left(f_{p}-{\tilde{f}}_{p}\right)}^{2}},\\ PSNR=10\cdot \log_{10}\left(\frac{\max^{2}\left(f\right)}{{RMSE}^{2}}\right), \end{array}$$

where *f* and $\tilde{f}$ are the reconstructed image and reference image, respectively, and *N* is the number of total pixels. The image quality assessment (IQA) index universal quality index (UQI) (the closer to 1, the higher image quality) [38] is also used for quality evaluation. Entropy of the reconstructed CT image is used for subjective evaluation since the true reference image is unknown and the reference image used in the following experiments is noisy. Smaller entropy corresponds to images with weaker noise and artifacts [39].

The first concern focuses on the number of projection views, which is a significant factor for the temporal resolution. Thus, projections at 36, 60, and 84 views from the 3D Shepp-Logan phantom are used for CT reconstruction to study the influence of the number of projection views, respectively. We stress that this manuscript is to prove the feasibility of the MCCT using TV and TF-L0 methods. From Table 1, we can see that both TV and TF-L0 can acquire high image quality using 60 and 84 projection views. The reference image is the phantom itself. The good performance of TV and TF-L0 can be explained as follows. The 3D Shepp-Logan phantom is sparse in the gradient domain, which is coincident with the assumption of the TV method. However TV minimization would deliver to equal punishments for all the image gradients, which may blur the edges. The 3D Shepp-Logan phantom is also sparse after tensor framelet transform. From (7), we can see that TF-L0 penalizes the L0 norm of the coefficients *Wf*, which will not penalize the large coefficients; thus, the edge information can be effectively retained. The quantitative image metrics in Table 1 show that TF-L0 performs the best in this experiment. From the experiment results, we can see that 60 projection views may be a good choice to balance the image quality and the temporal resolution. Based on the observation, only 60 projection views are used to inspect the performance of the proposed MCCT in the following section.

The second concern focuses on the ability of noise suppression. We add Gaussian noise to the 60 noise-free projections of the 3D Shepp-Logan phantom. The mean value of Gaussian noise is 0. The standard deviation is 0.1% and 0.3% of the maximum value of the projection data, respectively. Then, the quantitative image metrics of the images are listed in Table 2. We can see that both TV and TF-L0 can acquire higher quantitative image metrics in this experiment. TF-L0 also performs the best. Based on the experiments, we can see that TV and TF-L0 have great potential to reconstruct high-quality images from sparse noisy projections. So, TV and TF-L0 are used for image reconstruction of the MCCT system in the following sections.

### 3.1. Simulate Dynamic Object Reconstruction for MCCT

The purpose of this presented MCCT is to improve the temporal resolution. We modified the volume data of buckwheat seed reconstructed from real CT projections acquired from a circular cone-beam micro-CT in the Engineering Research Centre of Industrial Computed Tomography Nondestructive Testing of the Ministry of Education of China to simulate the dynamic process. Then, the simulated projections can be calculated in the imaging geometry constituted by the MCCT. Three experiments are designed to verify our idea. For convenience of narration, the identifiers of these experiments are set to 1, 2, and 3, respectively.

**Figure 6:**
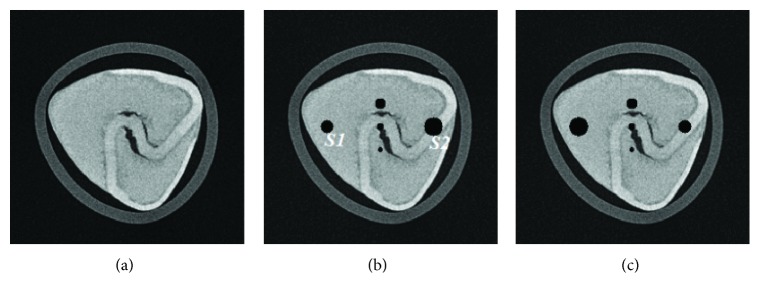
(a) The original buckwheat seed image; (b and c) initial and final state, the modified buckwheat seed image with two changing spheres each of which changes smoothly in size.

Because the original object is static, two spheres each of which changes smoothly in size are inserted into the object to simulate the motions. Three other static spheres are also inserted into the object. The original and modified images are shown in Figure 6. Assume that all reconstructed images consist of 256 × 256 × 256 voxels with a voxel size of 0.078 × 0.078 × 0.078 mm^3^. Denote *r*_1_ as the radius of the smaller sphere *S*1 and *r*_2_ the radius of the larger sphere *S*2. Ultrafast scanning can collect consistent projection data because the dynamic object is considered to be “static” in an instant. Small changes of the spheres are used to simulate the motions of the object. In experiment 1, *r*_1_^2^ changes evenly from 0.5515 × 0.5515 mm^2^ to 0.7839 × 0.7839 mm^2^, while *r*_2_^2^ changes evenly from 0.7839 × 0.7839 mm^2^ to 0.5515 × 0.5515 mm^2^. Then, we acquire 60 different datasets to generate the projection data. To simulate smaller changes in experiment 2, *r*_1_^2^ changes evenly from 0.5515 × 0.5515 mm^2^ to 0.6777 × 0.6777 mm^2^ and *r*_2_^2^ changes from 0.7839 × 0.7839 mm^2^ to 0.6777 × 0.6777 mm^2^. We also acquire 60 different datasets to generate the projection data. The projection data at 60 projection views are also inconsistent with each other but the degree of inconsistency is less than that in experiment 1. In experiment 3, the static modified buckwheat seed to be scanned is the fifteenth frame of the changing modified buckwheat seed in experiment 1. Then, consistent projection data are available to conduct CT reconstruction.

**Figure 7:**
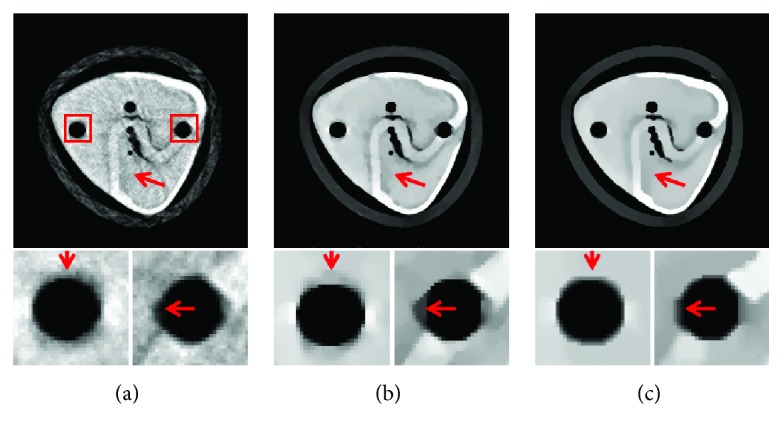
CT images of the modified buckwheat seed in experiment 1: (a) SART, (b) TV, and (c) TF-L0. The second row images are the close-ups of ROIs. The places pointed out by red arrows are examples where TF-L0 acquires the best results.

In experiment 1, 60 projection views are used to inspect the performance of the proposed MCCT. Each projection view corresponds to one dataset. Our goal is to reconstruct the CT images at the beginning instant of the CT scan. The reference image is the initial image. The quantitative analysis below is performed using this reference image with some noise. The images are reconstructed using (i) SART, (ii) TV, and (iii) TF-L0. Reconstructed images are shown in Figure 7, where two ROIs are selected to clearly show the reconstruction quality of SART, TV, and TF-L0. Quantitative indexes RMSE, PSNR, and UQI in Table 3 show the effectiveness of the TV and TF-L0 methods. Because the real reference image is unknown and the reference image used here suffers from noise, the subjective evaluation index “entropy” is also calculated for comparison. The smaller entropy of the images from TF-L0 shows that TF-L0 can acquire CT images with weaker noise or artifacts. Two spheres *S*1 and *S*2 have not been reconstructed accurately for all reconstruction methods because of the data inconsistency.

**Figure 8:**
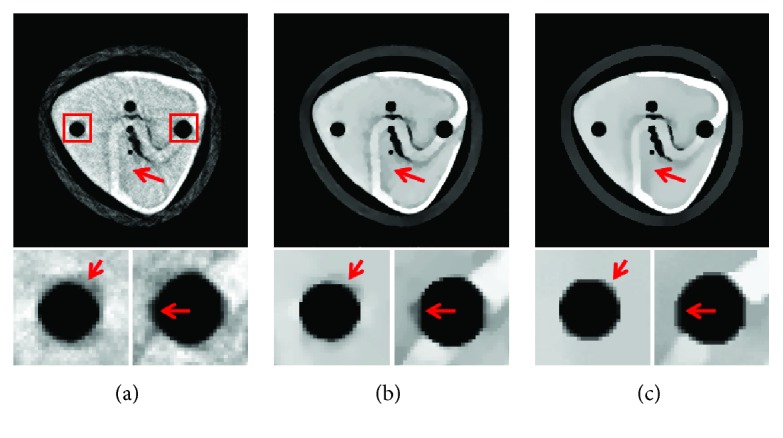
CT images of the modified buckwheat seed in experiment 2: (a) SART, (b) TV, and (c) TF-L0. The second row images are the close-ups of ROIs. The places pointed out by red arrows are examples where TF-L0 acquires the best results.

**Figure 9:**
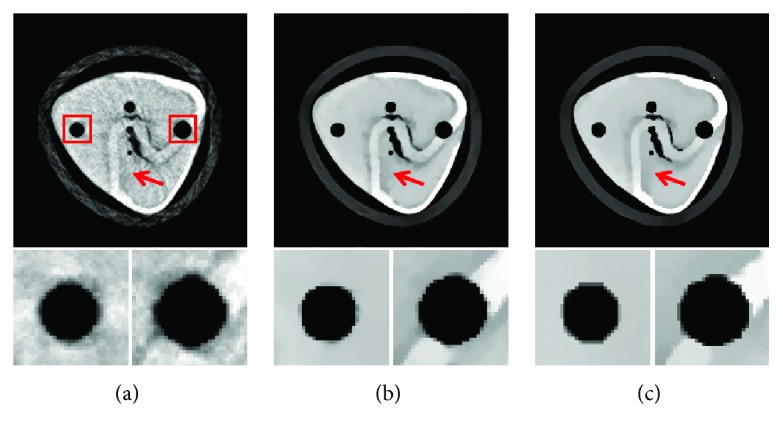
CT images of the modified buckwheat seed in experiment 3: (a) SART, (b) TV, and (c) TF-L0. The second row images are the close-ups of ROIs.

The main purpose of the presented MCCT is to improve the temporal resolution. But the improvement cannot be seen from the results in experiment 1 due to the motions. To show the improvement of the image quality, the modified buckwheat seed with smaller changes and the static modified buckwheat seed are simulated in experiment 2 and experiment 3, respectively. The aim is to show that combining with the reconstruction methods TV and TF-L0 the MCCT can acquire decent image quality. Only in this way, can we draw the conclusion that the MCCT can balance the image quality and the temporal resolution. For experiment 2 and experiment 3, images are reconstructed by (i) SART, (ii) TV, and (iii) TF-L0. Reconstructed images are shown in Figures 8 and 9, respectively. The RMSE, PSNR, UQI, and “entropy” indexes of these results are also listed in Table 3. From these results, we can see that the effectiveness of TF-L0 is not obvious compared to TV; but the smaller entropy of the image from TF-L0 shows that TF-L0 can reconstruct CT images with less artifacts. Two spheres *S*1 and *S*2 have been reconstructed with higher accuracy in experiments 2 and 3 than in experiment 1.

From the quantitative image metrics in Table 3, we can see that TV performs better than TF-L0 in terms of RMSE, PSNR, and UQI; TF-L0 performs better than TV in terms of entropy. Since the real reference image is unknown and the reference image is noisy, the quantitative image metrics RMSE, PSNR, and UQI may only roughly depict the image quality. From the images in Figures 7–9, we can see that both TV and TF-L0 can reconstruct high-quality images. When comparing TV and TF-L0, we find that they can acquire similar image quality, but they show different efficacies in terms of reducing the artifacts. For the TV method, it tends to produce a CT image with a piecewise constant property. The TV method may produce some blocky artifacts (Figures 7(b), 8(b) and 9(b)) since the buckwheat seed does not have the piecewise constant property. For TF-L0 method, since the piecewise-linear framelet basis, which involves high-order derivatives, is used in this experiment, images reconstructed by the TF-L0 method are free from the blocky artifacts. Besides, L0-norm minimization of the coefficients would not penalize the large coefficients; thus, the edge information can be effectively retained (the second row images in Figures 7–9).

**Figure 10:**
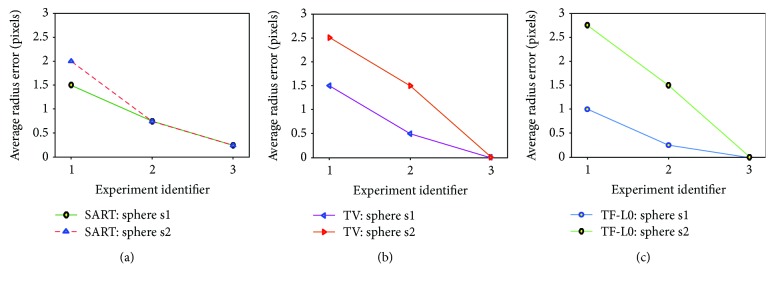
Data analyses of experiments 1, 2, and 3 are performed to show the influence of the motions. Smaller average radius error represents higher image quality.

To see the reconstruction accuracy intuitively, simple data analyses from these three experiments are performed to show the influence of the motions on the image quality. In general, the motion-induced artifacts would blur the image edges. Thus, the accuracy of the edges of these two reconstructed spheres can be used to assess the influence of the motions. Because the reference image is available at each instant, the average radius errors can be calculated and then plotted in Figure 10 for experiments 1, 2, and 3. From the results, we can see that the smaller the degree of data inconsistency, the higher is the acquired reconstruction accuracy. This conclusion seems to be obvious, but in practice it is difficult to acquire consistent data. That is why we propose the MCCT system. Based on these experiment results, we can conclude that combining with the developed TF-L0 method or other potential reconstruction algorithms such as TV, the MCCT has the potential to balance the image quality and the temporal resolution.

**Figure 11:**
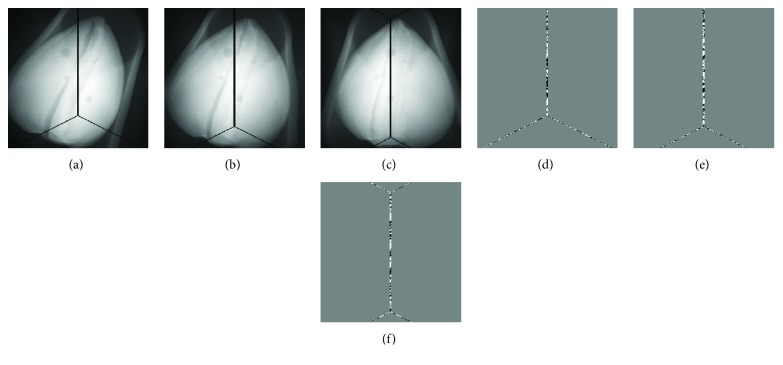
Three typical types of projection data loss: (a, b, c) the second images are the images showing the differences between the ideal projection and the interpolated projection.

**Figure 12:**
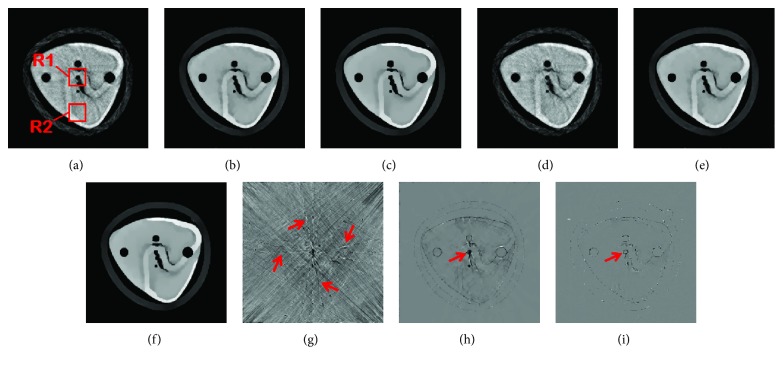
CT images from SART (a, d, g), TV (b, e, h), and TF-L0 (c, f, i). Images (a), (b), and (c) are reconstructed from the ideal projections. Images (d), (e), and (f) are reconstructed from the interpolated projections. The third row images are the images showing the differences between images in the first and the second rows.

In the experiments above, the projection data without data loss are used for CT reconstruction. Actually, the projection data loss is inevitable because of the gaps caused by the X-ray tube or the X-ray exit windows. Without loss of generality, we assume that the gaps result in a three-pixel-wide data loss on the virtual detector. Considering the case of 60 focal spots, we analyze the types of the projection data loss and divide them into three types as shown in Figure 11. The missing data are recovered by linear interpolation. The images reconstructed from the ideal projections and the interpolated projections are shown in Figure 12. From the images reconstructed by SART, we can see that the data loss can cause some stripe-like artifacts diverging from the image center (Figure 12(g)). That is to say, the center part of the image loses more information due to the data loss. For TV and TF-L0, these artifacts are effectively removed. From the difference images in Figures 12(g), 12(h), and 12(i), we can see that TF-L0 can reduce furthest the degradation of the image quality caused by data loss. In order to objectively assess the degradation of the image quality, we define the change rate (CR) of the image quality as follows:

(15) $$\begin{array}{c} CR\left(IQA\right)=\frac{\left|{IQA}_{ideal}-{IQA}_{interpolation}\right|}{{IQA}_{ideal}}\times 100\%, \end{array}$$

where IQA can be replaced by RMSE, PSNR, and UQI. IQA_ideal_ represents the image metric of the image reconstructed from the ideal projections, while IQA_interpolation_ represents the image metric of the image reconstructed from the interpolated projections. Then, the CR with regard to RMSE, PSNR, and UQI are listed in Table 4. Besides, we select two regions R1 and R2 (pointed out in Figure 12(a)) to show the impact of the data loss on the image quality. The region R1 is located in the center of the image, while the region R2 is located in the lower part of the image. From Table 4, we can see that for each reconstruction method, the CR of region R1 is the largest one, which indicates that the center region R1 is more affected by the projection data loss. For the full image, R1 and R2, we can conclude that TF-L0 can acquire the smallest CR; that is to say, compared to SART and TV, TF-L0 is more robust to the projection data loss. In general, based on the sparsity in the gradient domain or the tensor framelet transform domain, TV and TF-L0 can reduce the impact of the data loss on image quality. In conclusion, TF-L0 performs the best in terms of dealing with the projection data loss.

### 3.2. Simulate Dynamic Object Reconstruction for MCCT Using Bee Dataset

**Figure 13:**
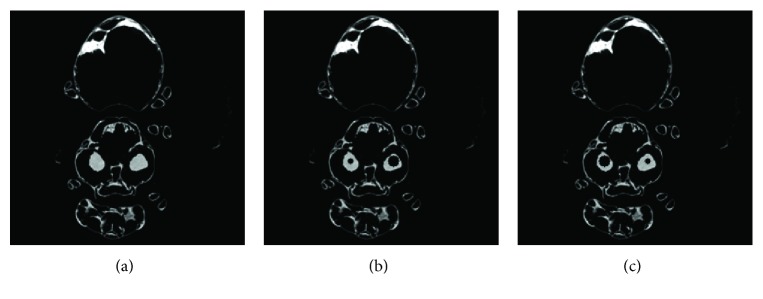
(a) The original bee image. (b, c) Initial and final state; the modified bee image with two spheres each of which changes smoothly in size.

In order to simulate the real situation more realistically, an arthropod animal bee dataset is used to simulate the dynamic procedure. Similarly, two spheres are inserted into the bee data set. One sphere becomes larger; the other becomes smaller (Figure 13).

**Figure 14:**
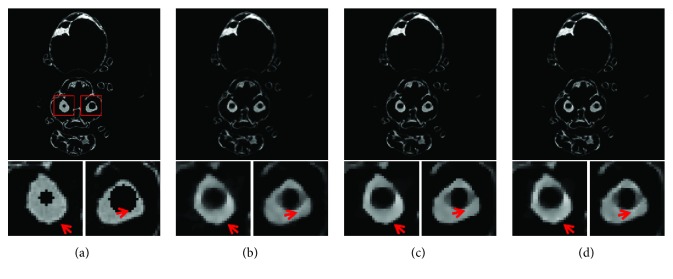
CT images of the modified bee reconstructed from inconsistent projections: (a) the reference image; (b) SART; (c) TV; (d) TF-L0. The second row images are the close-ups of ROIs. The places pointed out by red arrows are examples where TV and TF-L0 acquire different results.

**Figure 15:**
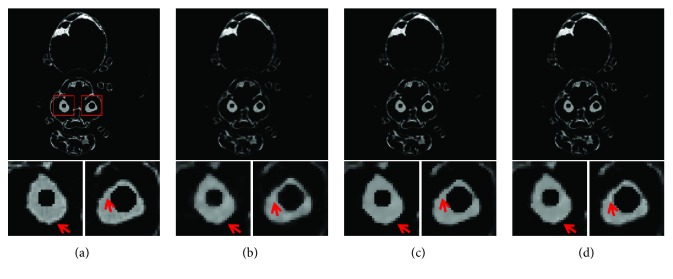
CT images of the modified bee reconstructed from consistent projections: (a) the reference image; (b) SART; (c) TV; (d) TF-L0. The second row images are the close-ups of ROIs. The places pointed out by red arrows are examples where TV and TF-L0 acquire different results.

In the dynamic procedure, 60 different datasets are used to generate projections. The reconstructed CT images are shown in Figure 14 using inconsistent projections. These two spheres have not been reconstructed accurately for all reconstruction methods because of the data inconsistency. Then, the fifteen datasets in this dynamic procedure as a static object is used to acquire consistent projections for CT reconstruction. The final CT images are shown in Figure 15. To further suppress the noise in the reconstructed images, bilateral filtering is performed on all the reconstructed images in this section. From visual inspection, the CT image qualities acquired by TV and TF-L0 are similar to each other. RMSE, PSNR, UQI, and the “entropy” of these results are shown in Table 5 for dynamic and static reconstruction. From these results, we can see that TV and TF-L0 can acquire a higher image quality. From Table 5, we can see that the difference between TV and TF-L0 is also small. These results show that TV and TF-L0 have the potential to acquire a decent image quality using projections with weaker data inconsistency. This conclusion seems obvious, but the difficulty is how to acquire consistent projections. That is why we propose this MCCT system.

In this experiment, the bee specimen has complex structures. The reconstructed images show different results from the images in Section 3.1. From the quantitative image metrics in Table 5, we can see that TV and TF-L0 can reconstruct similar image qualities. However, from the reconstructed images (Figures 14 and 15) we can see that TV and TF-L0 show different efficacies in terms of image smoothing and edge preserving. For the TV method, it tends to reconstruct a highly smooth image (the images below Figures 14(c) and 15(c)) since the TV minimization penalizes all image gradients equally. Thus, the edges may be overly smoothed. TF-L0 can also acquire smooth images by indirectly penalizing the coefficients *Wf*. This indirect operation on image smoothing can reduce most of the artifacts, but some residual artifacts may remain. We can see that there are some residual artifacts (the images below Figures 14(d) and 15(d)) on the images reconstructed by TF-L0, but the edges shown in the close-ups are closer to that in the reference image.

## 4. Conclusions

The proposed MCCT possesses the cube structure with CNT-based X-ray tubes. The CNT-based X-ray source has a compact structure because multiple electron emission points can be integrated into one cathode. The miniaturization of the X-ray source makes it possible to design the MCCT system with some advantages. In short, the cube shape avoids the conflicts between the X-ray and the detector without restricting the size of the FOV. Mechanical rotation is replaced by selecting different X-ray focal spots. Approximately sparse sampling, little response time, and electronic programmability of the CNT-based X-ray focal spots improve the temporal resolution. Furthermore, the MCCT system only works for some small dynamic objects; system parameters can be optimized to balance the temporal resolution and the image quality. The MCCT is an initial proof-of-concept design which helps us to understand the difficulties of the system design.

First, the possible drawback is that the special sampling on the twelve edges may lead to some artifacts looking like limited-angle artifacts when the positions of the electron emission points are not spread evenly. To deal with this problem, we can more evenly spread these electron emission points. The CNT-based X-ray tube mainly includes two parts: an electronic gun for installing the field emission cathode and the vacuum chamber for the encapsulation of the electronic gun. The second problem is that the gaps (Figure 1) between two detectors may be a major problem in terms of data completeness and reconstruction, since vacuum chambers may be large. Thus, some projection data are missing. To deal with this problem, the designed X-ray exit windows are located in the virtual edge of the cube to reduce the room occupied by the chambers as much as possible. Inspired by [23], the missing projection data can be compensated to a certain extent through linear interpolation. Third, because the detectors collect photons from different fixed focal spots in the MCCT, there is no antiscatter grid at the detectors. The focal spots are also activated in sequence, so there is no cross-scatter. However, the forward scatter still makes it difficult to reconstruct an accurate image. Similar to the work in [21], the forward scatter can be measured and addressed by the scatter correction method. Fourth, based on the quest for the cardiac CT scanner in [7], the system cost may be another problem. Multiple X-ray sources and detectors currently make the MCCT system expensive. As CT technologies evolve, some current problems may be addressed.

From the experiments in Sections 3.1 and 3.2, we can see that TV and TF-L0 can acquire similar high-quality images. When comparing TV and TF-L0, we can see that TV and TF-L0 show different efficacies in terms of reducing the artifacts, image smoothing, and edge preserving. These different efficacies come from different prior assumptions on CT images. For the TV method, it assumes that a CT image is sparse in the gradient domain. TV minimization would deliver to equal punishment on all image gradients. So the TV method tends to produce a smooth image with a piecewise constant property, which in turn would smooth the edges or introduce some blocky artifacts. For the TF-L0 method, it assumes that a CT image can be sparsely represented by the framelet basis functions. Then, L0 minimization of the framelet transform coefficients can discard some small coefficients which usually come from noise or artifacts. TF-L0 would not penalize the large coefficients; thus, the edges can be well retained though this indirect way to regularize the image while some residual artifacts may remain. These results have proven the feasibility of the MCCT. Reconstruction results show that 60 focal spots may be a good choice to balance the image quality and the temporal resolution. In practice, the number of the projection views can be further optimized. The MCCT system is proposed for dynamic objects. Therefore, a series of reconstructed images can be acquired by successive scanning and reconstruction. With the development of 4D image reconstruction methods, fewer projection views may be needed for each frame of the dynamic object, which is propitious to improve temporal resolution. We will develop this reconstruction method for the MCCT in the follow-up work. The long-term goal of this research is to estimate the radiation dose from the MCCT scan protocol and build a real MCCT system. As we know, rabbit and mouse models mimicking human physiology and pathology are popular in biomedical research. Thus, estimating the radiation dose is significant for some small animals. These results will enable us to further improve the MCCT system.

In conclusion, we propose the MCCT system and verify its feasibility; three reconstruction methods are used to prove its feasibility. The simulation results show that the proposed MCCT has the potential to improve the temporal resolution. Based on the conclusion and the advances in X-ray source and detector technology, the proposed MCCT is expected to have the potential practical merits.

## Figures and Tables

**Table 1:** RMSE (10^−3^), PSNR, and UQI of the reconstructed Shepp-Logan using noise-free projections.

Views	RMSE			PSNR			UQI		
	SART	TV	TF-L0	SART	TV	TF-L0	SART	TV	TF-L0
36	41.68	30.14	**23.36**	27.60	30.42	**32.63**	0.9747	0.9858	**0.9922**
60	27.04	15.76	**2.23**	31.36	36.05	**53.04**	0.9896	0.9965	**0.9999**
84	22.83	13.27	**0.93**	32.83	37.54	**60.61**	0.9926	0.9975	**0.9999**

**Table 2:** RMSE (10^−3^), PSNR, and UQI of the reconstructed Shepp-Logan using 60 noisy projections.

	RMSE			PSNR			UQI		
	SART	TV	TF-L0	SART	TV	TF-L0	SART	TV	TF-L0
Noise free	27.04	15.76	**2.23**	31.36	36.05	**53.04**	0.9896	0.9965	**0.9999**
0.1% noise	27.26	16.21	**2.37**	31.29	35.80	**52.50**	0.9894	0.9963	**0.9999**
0.3% noise	28.98	19.76	**2.69**	30.76	34.08	**51.40**	0.9880	0.9944	**0.9999**

**Table 3:** IQAs of the reconstructed buckwheat seed in experiment 1, experiment 2, and experiment 3, respectively (unit of RMSE: 10^−3^).

Experiment	1			2			3		
	SART	TV	TF-L0	SART	TV	TF-L0	SART	TV	TF-L0
RMSE	43.37	**29.19**	31.42	42.49	**28.68**	31.1	42.05	**28.30**	30.47
PSNR	27.25	**30.70**	30.06	27.43	**30.85**	30.14	27.52	**30.96**	30.32
UQI	0.9758	**0.9890**	0.9873	0.9768	**0.9894**	0.9876	0.9772	**0.9897**	0.9881
Entropy	6.57	4.80	**4.47**	6.55	4.74	**4.47**	6.53	4.72	**4.50**

**Table 4:** Change rate of the image metrics of the images reconstructed from the interpolated projections.

Region	Full image			R1			R2		
Change rate	SART	TV	TF-L0	SART	TV	TF-L0	SART	TV	TF-L0
RMSE	1.78%	2.01%	**0.76%**	7.51%	12.9%	**3.60%**	2.06%	1.58%	**0.85%**
PSNR	0.59%	0.58%	**0.23%**	3.38%	4.45%	**1.34%**	0.66%	0.50%	**0.28%**
UQI	0.06%	0.03%	**0.01%**	2.75%	1.28%	**0.51%**	0.42%	0.08%	**0.05%**

**Table 5:** IQAs of the reconstructed bee images (unit of RMSE: 10^−3^).

	Dynamic			Static		
	SART	TV	TF-L0	SART	TV	TF-L0
RMSE	4.81	3.98	**3.77**	4.67	**3.36**	3.57
PSNR	47.73	49.39	**49.86**	48.00	**50.84**	50.32
UQI	0.9482	0.9654	**0.9691**	0.9513	**0.9759**	0.9723
Entropy	1.98	1.29	**0.88**	1.98	1.61	**0.86**

## Data Availability

The data used to support the findings of this study were supplied by Engineering Research Centre of Industrial Computed Tomography Nondestructive Testing of the Ministry of Education of China under license and so cannot be made freely available. Requests for access to these data should be made to Chongqing Zhence Science and Technology Company Limited (http://www.meansee.com/).
